# Engaging otherness: care ethics radical perspectives on empathy

**DOI:** 10.1007/s11019-023-10152-0

**Published:** 2023-05-12

**Authors:** Jolanda van Dijke, Inge van Nistelrooij, Pien Bos, Joachim Duyndam

**Affiliations:** 1 University of Humanistic Studies, Kromme Nieuwegracht 29, Utrecht, 3512 HD The Netherlands; 2 Care Ethics, University of Humanistic Studies, Utrecht, The Netherlands; 3 Research Methodology, University of Humanistic Studies, Utrecht, The Netherlands; 4 Philosophy, University of Humanistic Studies, Utrecht, The Netherlands

**Keywords:** Care ethics, Radical empathy, Empathic bias, Otherness, Presence theory, Engrossment, Empathy education

## Abstract

Throughout the years, care ethicists have raised concerns that prevalent definitions of empathy fail to adequately address the problem of otherness. They have proposed alternative conceptualizations of empathy that aim to acknowledge individual differences, help to extend care beyond one’s inner circle, and develop a critical awareness of biases and prejudices. We explore three such alternatives: Noddings’ concept of engrossment, Meyers’ account of broad empathy, and Baart’s concept of perspective-shifting. Based on these accounts, we explain that care ethics promotes a conceptualization of empathy that is radical in its commitment to engage otherness and that is characterized by being: (1) receptive and open, (2) broad and deep in scope, (3) relational and interactive, (4) mature and multifaceted, (5) critical and reflective, (6) disruptive and transformative. This type of empathy is both demanding and rewarding, as it may inspire health professionals to rethink empathy, its challenges, and its contribution to good care and as it may enrich empathy education and professional empathy practices in health care.

## Introduction

This paper explores care ethics empathy, a distinctive form of empathy that is radical in its commitment to engage otherness and acknowledge individual differences between people. While empathy has traditionally been understood as a multidimensional concept that can have affective and cognitive aspects (Hojat et al. 2002), in the field of health care it is typically conceptualized as the cognitive capacity to understand the other’s experiences, combined with the ability to communicate this empathic understanding to the patient (Bearman et al. 2015). Hojat (2016) provides an example of this prevalent definition:

Empathy is a predominantly cognitive (rather than an affective or emotional) attribute that involves an understanding (rather than feeling) of experiences, concerns, and perspectives of the patient, combined with a capacity to communicate this understanding, and an intention to help (p. 74).

In health care, affective or emotional empathy is considered analogous to sympathy (Hojat 2016) and involves “the capacity to enter into or join the experiences and feelings of another person” (Hojat 2002, p. 1563). One of the reasons for the distinction between empathy and sympathy in health care is that the affective aspects are considered to have undesirable outcomes in patient care, as emotions may interfere with the health professional’s clinical objectivity and effectiveness (Hojat 2016, p. 14). A cognitive understanding of empathy is in line with a form of professionalism described as “detached concern” (Jeffrey 2016).

The significance of empathy in health care is widely acknowledged throughout the field including but not limited to oncology, palliative care, general practitioner care, geriatrics, mental health care, and emergency care. In the emergency room, for example, empathy helps to establish so-called “emergency rapport”, a working alliance between the emergency physician and the patient (Rosenzweig 1993). Empathy is crucial in medical practice as it creates a safe and non-judgmental space that encourages patients to be open and honest with their healthcare providers. This is particularly important in the emergency room, where patients may withhold vital information they feel ashamed or embarrassed about, such as illicit drug use or risky sexual behaviors, which can have a detrimental effect on their medical care (Long 2017). In oncology, patients often struggle to retain complex information about treatment options and their potential side effects due to high emotional stress, which can impede patient-centered decision-making and care (Westendorp et al. 2021). Research into clinician-expressed empathy suggests that empathy leads to improved information recall in (advanced) cancer patients, as it is a powerful way to reduce emotional distress (Westendorp et al. 2021).

Despite empathy being essential in health care, research indicates that empathy levels among health professionals tend to be low and that empathy erodes during medical and nursing education (Hojat 2016; Hojat et al. 2004; Jeffrey 2016; Marcus 1999; Pedersen 2010; Perrella 2016; Shapiro 2008; Ward et al. 2012). Various reasons have been cited for this decline, including the dominance of the biomedical model, the rise of technology and computer-based diagnostics, and a lack of time and patient interaction (Hojat 2016). While empathy education is a key means to enhance empathy in health professionals, current methods of empathy training have been criticized (Perrella 2016). In line with empathy’s definition as a predominantly cognitive construct, empathy education tends to focus on teaching a selective set of skills, particularly cognitive perspective-taking skills, empathic communication skills, and behavioral strategies (Perrella 2016). Less attention has been given to affective empathy and emotion regulation or they may even be absent in empathy education (Ekman and Halpern 2015).

Another limitation is that simulation-based empathy education is increasingly becoming the norm to train empathy in health professions (Bearman et al. 2015; Wear and Varley 2008). Role-playing and perspective-taking exercises are among the most common forms of simulation-based empathy education (Englander and Folkesson 2014). For example, to train and evaluate the student’s degree of empathic behavior and communication, empathy education often draws on role-playing exercises with standardized patients (Berg et al. 2015; Perrella 2016; Wear and Varley 2008). This type of empathy education has significant benefits as it provides a safe environment to practice empathy, but critics warn of its limitations. The lack of interaction with real patients and the risk of reducing empathy to a communication strategy or learned behavior are among their concerns (Englander, 2014; Hanna and Fins 2006; Shapiro 2008). There has been a call within health care to enhance empathy education programs by encouraging students to interact with real patients, engage with lived experiences and foster humanistic skills (Hanna and Fins 2006; Perrella 2016).

In this paper, we explore care ethics empathy. The care ethics perspective is in line with those authors in the field of health care who are concerned about empathy decline and are seeking means to enhance empathy practice and education in this field. Care ethics is a political and moral theory that has care practices as one of its main topics of interest and that is characterized by: (1) A relational anthropology as opposed to an anthropology based on autonomy; (2) A multidimensional, practice-based epistemology that values emotions and a variety of alternative knowledge sources, rather than being limited to reason; (3) A form of moral deliberation that values insights from multiple perspectives and that is primarily based on particularism and contextuality as opposed to being founded on universal, abstract, and general principles; (4) A sensitivity to power relations and the aim to include and give a voice to people who are dependent and socially excluded (Sander-Staudt 2011).

In general, care ethicists are keenly aware of both the moral significance and disadvantages of empathy (Hamington 2017). From a care ethics perspective, empathy is not inherently good and is in itself not sufficient to guarantee good care. While care ethicists recognize that empathy can have important moral functions, they also acknowledge empathy’s limitations, particularly regarding otherness (Van Dijke et al. 2019). The term “otherness” has many different connotations across different disciplines. In empathy literature, it has a long and complex history. Based on the care ethics perspective, we focus on a primarily political interpretation of the term “otherness.” In this context, the problem of otherness refers to the fundamental question of how to acknowledge and respect differences between people and how to include people who are perceived as “other” or different. Acknowledging otherness and extending care, empathy, and sympathy to people who are different and who may be vulnerable and marginalized, are among the most urgent topics in the field of care ethics (Koehn 1998; Tronto 1993). Empathy is particularly challenged in relation to people who are perceived as “other” or different from the norm, or who went through experiences that one has not (yet) been exposed to, such as grief, trauma, depression, or psychosis (Pienkos and Sass 2012; Ratcliffe 2012, 2014, 2017).

Care ethicists have addressed at least two problems in relation to empathy and otherness. First, they point out the risk of self-referential accounts of empathy, particularly projection and self-focused perspective-taking, as these accounts may fail to do justice to otherness (Meyers 1994; Noddings 2010a). Self-focused perspective-taking means that people try to mentally reconstruct the other’s experiences by projecting themselves and their own personality, values, and needs into the other’s circumstances, trying to imagine “what it would be like” to be in that position (Meyers 1994, 2004). Care ethicists consider self-referential or projective empathy accounts to be problematic (Noddings 1984/2013). As Ruddick (1989) argues, “the idea of empathy, as it is popularly understood, underestimates the importance of knowing another without finding yourself in her” (p. 121). When people use their own experiences and characteristics as a frame of reference to empathize with others, they tend to overlook crucial differences, fail to adequately understand others’ concerns, or are unable to respond with the appropriate emotion to what people are going through. In the field of health care, self-focused perspective-taking can lead to care that is out of tune with clients’ individual experiences and with what they actually value and need. The problem of self-referentiality has been one of the main reasons why care ethicists have proposed alternative conceptualizations, such as engrossment.

Second, empathy can be diminished because of biases and prejudices towards those who are different or “other” (Meyers 1994; Noddings 2010a, b; Slote 2007; Tronto 1993). A well-known example of this problem is the familiarity bias. Research indicates that people empathize more readily and accurately with people who are perceived as familiar and similar or who are part of the same ingroup (Hoffman 2001; Oxley 2011). People who seem unfamiliar risk being identified as belonging to a different group (e.g., an outgroup) and being socially excluded, a problem known as “othering” (Tronto 1993). Empathic biases can be perpetuated and exacerbated by prejudices (Meyers 1994). Those belonging to a minority group often face being devalued or excluded by the dominant social group based on certain characteristics, such as sexual orientation, gender, religion, or skin color (FitzGerald and Hurst 2017; Fourie et al. 2017). In addition, they can be subjected to demeaning stereotypes that perpetuate the problem of “othering.” Since biases and prejudices tend to function on a subconscious level and are deeply ingrained in society, they are particularly difficult to recognize and deconstruct (Meyers 1994). Thus, both biases and prejudices pose serious obstacles to empathy (Meyers 1994; Oxley 2011).

While the empathy disadvantages that care ethicists identify have been noted across many disciplines throughout history, care ethicists have given significant weight to them, leading some care ethicists to reject the concept of empathy altogether and propose alternative conceptualizations. In this paper, we examine the viewpoints and empathy alternatives of three authors belonging to the care ethics field of inquiry. First, we discuss the concept of engrossment that was developed by care ethics pioneer Nel Noddings. Noddings argues that people need to empathize by attentively *receiving* the other instead of *projecting* themselves onto the other. She coins the concept of engrossment to refer to a receptive type of empathy that is deeply grounded in attentiveness. Second, feminist philosopher Diana Meyers proposes the concept of “broad empathy,” which expresses the idea that instead of empathizing with the other’s current situation, one has to empathize with the other’s life as a whole and thus gain a deeper insight into what it is like to actually be that other person. Third, Dutch care ethicist and empirical philosopher Andries Baart introduces the concept of “perspective-shifting” [translation by first author], which expresses the idea that to engage otherness and comprehend what values are at stake for people, professional caregivers need to shift from a detached outsider’s to an engaged insider’s perspective, ideally by being attentively present for their clients, by engaging in a dedicated caring relationship, and by exposing themselves with all of their senses to the client’s lifeworld. These three alternative conceptualizations to prevalent empathy accounts are at the heart of these authors’ respective moral and caring theories.

It is our hope that a care ethics perspective on empathy can enrich empathy’s conceptualization in the field of health care, help enhance and inspire empathy education, and address empathy’s disadvantages concerning the problem of otherness. At a time when health professionals encounter a very diverse group of patients, the commitment and ability to engage otherness and acknowledge biases and prejudices are of particular importance (Shapiro 2008). While some of the ideas that care ethicists propose are not new or exclusive, we argue that the care ethics perspective on empathy is, overall, distinctive in the field of health care. Care ethics empathy is radical in its commitment and ambition to thoroughly get to know the other *as other*, meet people on their own terms, acknowledge individual differences, and recognize and overcome biases and prejudices. It explicitly focuses on receptivity, is broad and multidimensional, involves a critical awareness of empathy’s limitations, and intends to challenge people’s views. Moreover, in line with the care ethics relational perspective, empathy is understood as a relational construct, which is unusual in health care where empathy is typically viewed as an individual ability (Van Dijke et al. 2020). In addition, since care ethicists are generally well aware of the limits and disadvantages of empathy, they acknowledge that empathy needs to be carefully integrated in care ethics or moral theory in such a way that its accompanying challenges and limitations can be accounted for. At the same time, this ambitious conceptualization of empathy poses a challenge in the field of health care, where empathy is often limited for a variety of reasons, a problem that we address in the discussion section.

In this paper, we first explore the three care ethics alternatives within the context of the authors’ broader moral and caring theories. Next, we describe the features of care ethics empathy and discuss both its challenges and its implications for empathy education in health professions. We focus on health professions such as nursing and medical care, as empathy and the problem of its decline are a topic of serious concern in these professions (Hojat 2016).

## Engrossment

Nel Noddings (*1929), an American mathematician, educationalist, and feminist philosopher, has been mentioned alongside Carol Gilligan as one of the “founding mothers” of care ethics (Hamington 2004). Although Gilligan introduced the term “ethics of care”, Noddings was the first to offer a comprehensive care ethics *theory* (Slote 2007). In *Caring* (1984/2013) she introduced the concept of engrossment, which lies at the core of her ethics of care.

Noddings criticizes principle-based ethics throughout her work. She coins the concept of “ethical caring” which seeks “to establish or restore natural caring” (Noddings 2010b, p. 37) and expand natural caring relationships to those beyond one’s immediate circle. Natural caring evolves from instinctive, maternal caring and represents Noddings’ ideal of good care (Noddings 1984/2013). She sees the dyadic bond between parent and child as the paradigmatic moral caring relationship. When caring comes naturally, people care for the other because they *want* to, not because they feel *obliged* to. Instead of consulting moral principles, they respond directly to the other’s needs (Noddings 1984/2013, p. xxiii). Noddings considers this type of caring to be pre-moral and pre-reflective, as natural caring neither requires moral effort nor moral thinking.

The natural inclination to care can, however, fail for various reasons. For example, it may fail because one is tired or because one finds the other unpleasant or even disgusting (Noddings 2005a). Another reason may be that the other does not belong to one’s empathic circle (Noddings 2010a, pp. 11–12). When this happens, Noddings argues that people can either resort to abstract principles and external rules, or they can turn to ethical caring (Noddings 2005a, 2010a). Unlike pre-moral natural caring, ethical caring requires moral effort and conscious moral decision-making. People ask themselves, “How would I respond if I were at my best caring self?” (Noddings 2010b, p. 68). Instead of a moral principle, natural caring and the ideal of one’s “caring-best” function as a moral compass guiding one’s actions. In ethical caring, people draw on their memories of natural caring, not only as “the one-caring” but also as “the one cared-for.” Noddings (1984/2013) argues that the yearning for caring and being in a caring relationship ultimately motivates people to act morally right.

The concept of engrossment is at the heart of natural caring. It is Noddings’ alternative to prevalent conceptualizations of empathy. She firmly rejects what she sees as the dominant, western, and “masculine” account that understands empathy as projecting oneself onto others and trying to rationalize or imagine what it must be like to be in the other person’s position (Noddings 1984/2013, 2010a, 2010b). According to Noddings, this empathy account is self-referential, overly rational, and indirect. She explicitly introduces engrossment as a feminine alternative in the first version of *Caring* (1984/2013).

Engrossment can be defined as “an open, nonselective receptivity to the cared-for” (Noddings 2005b, p. 15). By grounding empathy in receptive attention, the focus is directed towards the other’s situation, personality, needs, and experiences, rather than using one’s own experiences or characteristics as a frame of reference. Engrossment thus contributes to the outreaching, other-oriented movement of empathy, which discourages self-referential strategies.

Engrossment combines two elements: unbiased and non-judgmental receptive attention and affective empathy, or “feeling with” (Noddings 2010a). By using the concept of engrossment, Noddings emphasizes that people should not “invade,” but rather “be invaded” by the other, i.e., they should not project themselves but receive the other (Noddings 1984/2013). Engrossment is receptive, affective, and direct. It seeks to answer the question: “What are you going through?” (Noddings 2010b, p. 47) and refers to a direct way of receiving others’ needs. According to Noddings, mothers are naturally engrossed in their children. She gives the example of a child wetting itself. Mothers do not need to take the detour of cognitively analyzing the situation or projecting themselves onto their child to sense what is wrong. Instead, they directly and naturally receive and share their child’s feelings and needs (Noddings 1984/2013, p. 31).

Noddings emphasizes that receptive attention is non-judgmental and unbiased (Noddings 2010a, p. 9) and that it should be characterized by openness and vulnerability (Noddings 2012, p. 54). Receptive attention involves seeing the other in a positive light, which people are naturally inclined to do with those belonging to their empathic circle. If people fail to naturally see others positively, they can turn to ethical caring to identify, examine, and overcome their initial biases, prejudices, and judgments (Noddings 2010a, p. 11). Noddings cites an example provided by Iris Murdoch in *The Sovereignty of Good*, where a woman deeply dislikes her daughter-in-law but tries to see her in a more positive light (Noddings 2010a, b). She turns to ethical caring for guidance to overcome her initial aversion. The woman is prepared to care, invest in a caring relationship with her daughter-in-law, and reflect on her own narrow-mindedness. She realizes she is prejudiced and decides to look at her daughter-in-law with fresh eyes. Noddings explains: “From the perspective of care ethics, it is a matter of seeing the other in the best possible light. It means examining our own frame of mind and how it influences our understanding” (Noddings 2010a, p. 11).

Noddings (1984/2013) argues that, unlike cognitive or philosophical empathy, engrossment does not primarily serve an epistemic function, as it does not necessarily lead to knowledge claims or accuracy of understanding. Rather, its main moral functions are motivational and relational. Noddings’ phenomenological analysis suggests that the caregiver’s consciousness is characterized by two closely related elements: “engrossment” and “displacement of interest” or motivational displacement, which is a motivational energy directed toward others and their projects or needs (Noddings 2005b). The experience of engrossment is a deeply relational one. When caregivers are engrossed, they temporarily become “a duality” (Noddings 1984/2013, p. 30). They sense and perceive the situation both from their own perspective and that of the other: “I see through two pairs of eyes, hear with two sets of ears, feel the pain of the other self in addition to my own. My initial self is vulnerable, and it will be changed by this encounter” (Noddings 2002, p. 15). Noddings refers to this as a “dual perspective” (Noddings 1984/2013, p. 63). When people are thus engrossed, they begin to experience the other’s reality as a possibility for themselves, an experience that can change or transform them. The experience motivates them to act in accordance with this reality, for example by wanting to relieve the other’s pain or meet the other’s needs, thus acting altruistically. According to Noddings, this “displacement of interest” is the essence of caring. Therefore, all caring requires a certain level of engrossment (Noddings 1984/2013, p. 17).

The experience of being engrossed has the potential to change or disturb one’s ethical reality (p. 14). Noddings (1984/2013) illustrates this through an example of a colleague she used to have little regard for. She explains what happens when one day he tells her a touching story and she becomes engrossed in his experiences: “It is as though his eyes and mine have combined to look at the scene he describes… I feel what he says he felt. I have been invaded by this other” (p. 31). In turn, this dual-perspective sparks care and concern, which has a lasting impact: she is now fundamentally prepared to care. Noddings concludes she will “never again be completely without regard for him” (p. 31).

Eventually, Noddings discarded the term engrossment as it was frequently confused with infatuation, and as more hybrid types of empathy were gaining ground in empathy theory (Noddings 2010a, b). In recent times, she has used the concepts of “receptive attention” (Noddings 2002) and of empathy “with the understanding that it has both cognitive and emotional elements, and that the emotional element is primary” (Noddings 2010b, p. 12).

Noddings’ theory has been criticized for being “romantic” and politically naïve (Koehn 1998; Tronto 1993; Van Nistelrooij 2014). In contrast to Noddings’ affective account, Meyers (1994) presents an intellectual and political approach to empathy that focuses on people who are different, vulnerable, and marginalized. Meyers notes that these groups are often subjected to biases and prejudices, which seriously distort empathy and moral behavior. In her view, this urgent problem has been largely overlooked in moral theory and cannot be solved on an individual or relational level alone.

## Broad empathy

The American feminist philosopher Diana Meyers (*1947) co-edited the anthology *Women and Moral Theory* (1987), which remains a classic in the field of care ethics (Sander-Staudt 2011). With care ethicists, Meyers shares the critique of principle-based and universalist ethics, the appraisal of a feminine moral voice, the focus on moral relationships, and a keen interest in the topics of difference and othering (Koehn 1998; Oxley 2011). Her ethics is, however, more outspokenly feminist in her appraisal of women thinkers (Koehn 1998, p. 53) and her focus on the topics of social exclusion, cultural normative prejudices, and power (Oxley 2011). Her moral theory has been described as an ethics of empathy, as broad empathy is crucial to Meyers’ understanding of moral thought (Koehn 1998; Oxley 2011).

Meyers (1994, 2004) criticizes the dominant focus on impartial reasoning, principles, and rules that have prevailed in moral thought since the Age of Enlightenment. According to Meyers, this type of moral reasoning cannot do justice to differences and the complexity of everyday moral dilemmas. She proposes empathic thought as an alternative to a morality that relies on impartial reasoning. Empathic thought aims to build and maintain moral relationships with others and attune to their needs by asking the ultimate moral question “How can I *best* respond to you?” (p. 134). Empathic thought is the moral deliberation process that leads from empathic insight to moral decision-making. This type of moral thought or deliberation does not start from universal moral principles but rather springs from one’s moral identity. Moral subjects have their own moral ideals and sets of values, which they develop in relationships with others, based on mutual recognition and empathy. The fundamental question moral subjects ask themselves is: “Do you want to be the sort of person who would do such-and-such?” (Meyers 1994, p. 17). People contemplate what actions to take by considering both their own values and capacities (their moral identity) and their empathic understanding of the other’s values and needs (Meyers 1994, p. 17). Broad empathy is a key concept in Meyers’ moral theory because this kind of empathy is particularly suited to meeting others on their own terms, acknowledging differences, and gaining accurate empathic insights.

Like Noddings, Meyers (1994, 2004, 2016) rejects an understanding of empathy as predominantly projective and self-focused. She asserts that “empathy is defeated if one simply projects one’s own characteristic emotional responses onto the other” (Meyers 1994, p. 33), as it “does violence to other’s distinctive points of view” (Meyers 2016, p. 143). To prevent projection, Meyers proposes an understanding of empathy that is cognitive, other-focused, and broad in scope. She understands empathy as an imaginative reconstruction of the other’s experiences (Meyers  1994, pp. 125–126) that ideally arises from extensive interaction with the other (p. 37). She differentiates empathy from both “shrewdly sizing people up” and “sympathetically fusing with people” (p. 31). Meyers uses the term “sympathy” to refer to affective empathy (feeling with the other) and “empathy” to refer to the imaginative reconstruction of these feelings (p. 33). Citing Goldman (1992), Meyers argues that a vivid imaginative reconstruction of the other’s experiences is often affectively moving, but not to the extent that people can no longer differentiate between their own feelings and those of the other. Thus, they do not share the other’s subjective state.

In Meyers’ moral theory, empathy serves a variety of functions, including relational ones. According to Meyers, the primary moral function of empathy is epistemic, as the imaginative reconstruction aims to provide insight into the values that are at stake for the other. However, not all types of empathy are equally insightful. Meyers (1994) distinguishes between incident-specific and broad empathy, between “the most minimal incident-specific empathy with a stranger to the broadest empathy with an intimate” (p. 36). Incident-specific empathy is a narrow or focused type of empathy that aims to answer the question “What are you going through now?” (pp. 34–35), by trying to imagine what the other is experiencing in a given situation for a limited period of time. When people are unfamiliar with the other, they typically resort to self-focused, projective strategies: “Incident-specific empathy with strangers is accomplished by learning as much as one can about them and the situation they face, and then projecting as best one can one’s own profile of interests, needs, and the like into that constellation of circumstances” (p. 35). While Meyers (1994) notes that this self-focused form of empathy is the most common, its epistemic function is limited and possibly inaccurate. Incident-specific empathy can provide a general understanding of the other’s situation and experiences, but it does not reveal what those experiences *mean* to the other, what specific values are at stake, or how the situation affects that particular individual.

Meyers (1994) suggests that incident-specific empathy is (much) more accurate when practiced alongside broad empathy. This type of empathy is not restricted to a limited time or place, but instead, relies on a combination of imagination and an analysis of the other’s circumstances to reconstruct what it must be like to be that other person in the context of the other’s entire life and thus “empathize with another person’s subjectivity as a whole” (p. 35). To achieve this, broad empathy requires interaction with the other and concerned, receptive attention to various aspects of the other’s life (p. 37).

According to Meyers, broad empathy seeks to answer the question “What is it like to be you?” (p. 39). Thus, it is grounded in a more complex relationship with others than incident-specific empathy. In Meyers’ theory of empathic thought, the paradigmatic moral relationship is that between acquaintances since “this personalized model explains the subtlety and complexity we ordinarily associate with moral choice and action” (p. 136). While more intimate relationships, such as friendships, may provide more opportunities for interaction, being acquainted is a solid foundation for broad empathy since acquaintances typically know each other, interact with each other, and matter to each other to at least some degree (p. 136).

Broad empathy can help people reconsider their own values and moral identity, or even embrace new or different values and viewpoints, as “through empathy new constellations of needs, desires, beliefs, and values can be forged” (Meyers 1994, p. 38). This type of empathy has the power to change people’s ethical realities, broaden their horizons, and transform their value systems (Meyers 1994, 2016). Additionally, it can contribute to dismantling prejudices. However, broad empathy alone may not be sufficient to surmount persistent, cultural prejudices, as these often function at a subconscious level and are partly shaped, influenced, and sustained by one’s culture (Meyers 1994). Prejudices are perpetuated through cultural figurations such as stereotypes and can be exceedingly difficult to deconstruct. People may be emotionally invested in these prejudices and may genuinely believe that stereotypical images are part of an innocent tradition and are not harmful or oppressive (p. 54). Furthermore, prejudices can boost the self-esteem of members who belong to the dominant group (p. 11). Even those who are victimized by prejudices may defend them, as they are part of their worldview.

Meyers (1994) contends that prejudices can be surmounted through dissident speech, which she defines as “the activity of giving benign figurative expression to nonconscious materials that would otherwise distort moral judgment” (p. 59). Dissident speech aims to bring awareness to prejudicial stories and images, refigure them, and help revalue socially excluded groups or individuals. To overcome prejudices, people need to view others in a different light (p. 60). An important way to undermine prejudices is by developing “fresh figurations” or “emancipatory counterfigurations” (p. 60). These figurations are created in solidarity with marginalized people or are developed by minority groups themselves. As an example, Meyers cites the counterimage of androgyny introduced by feminists to subvert dominant gender norms (p. 69). Meyers argues that empathy can help evaluate the effectiveness of these counterfigurations. By empathizing with figurations that one initially finds offensive, one’s views can be challenged or disrupted. If the counterfiguration indeed helps to empathize with marginalized groups, this means the figuration has been successful. Thus, coupled with dissident speech, empathy can help expose and dispel prejudices and support social inclusion. In turn, dissident speech can help to expand empathy as it “clears the way for empathy between members of different social groups” (p. 15).

The importance of Meyers’ perspective on empathy lies in her distinction between incident-specific and broad empathy, her empathy-centered moral theory, and her focus on the problem of difference and prejudices in relation to empathy. Rather than drawing on self-focused perspective-taking, broad empathy is primarily other-focused. It aims to comprehend the other’s perspective through, ideally, extensive interactions with the other and thoughtful attention to the other’s life as a whole.

Baart shares Meyers’ concern for vulnerable and socially excluded people, and for the harmful effects of biases and prejudices. While Meyers provides a primarily intellectual account, Baart takes an empirical approach that is grounded in everyday care practices. His concept of perspective-shifting provides a direct, primarily perception-based approach for engaging with others’ experiences.

## Perspective-shifting

Andries Baart (*1952) is a Dutch empirical philosopher and care ethicist. In 2001 he published his foundational study, *A theory of presence*, which resulted from a seven-year grounded-theory study into the practices of pastoral ministers working in disadvantaged urban neighborhoods. Baart considers the presence theory to be a care ethics theory (Baart 2017; Baart and Grypdonck 2008; Baart and Vosman 2011). Among its care ethics characteristics are the centrality of caring relationships, the focus on people who are vulnerable and socially excluded, and a care-based understanding of society and politics (Baart 2017).

The presence theory was developed amidst a changing care and welfare sector in the Netherlands, where the language, mentality, and market system quickly became more dominant (Van Heijst 2011). Presence encompasses a fundamental critique of these developments and of the current state of the Dutch care and welfare sector, which, according to Baart, has created its own trap by favoring autonomy over acknowledgment of dependency and by relying on technology instead of being attentive to the client’s needs (Baart and Carbo 2013). Based on ongoing empirical research into a diversity of care and welfare practices, Baart argues that professional caregivers are trapped in an overregulated healthcare environment (Baart and Carbo 2013; Baart et al. 2011; Peeters 2016). Compliance with externally imposed rules has become an indispensable characteristic of professionalism. Excessive regulation, however, erodes practical wisdom and obscures insight into the client’s values and needs and into the caregiver’s professional understanding of what needs to be done.

The presence approach – the presence theory as it is practiced in daily care – offers an alternative or a countermovement to mainstream care practice Baart (2006b). Presence can be defined as “a practice in which the caregiver attentively concerns himself with the other, thereby learns to see what is at stake for the other – from desires to fear – and in relation to that tries to understand what can be done in the particular situation.” (Baart 2004a, pp. 40–41) [translation by Klaver & Baart] (Klaver & Baart 2011, p. 312). The focus is on attentiveness and on building and maintaining caring relationships. The paradigmatic relationship of the presence theory is that of a professional friendship: presence practitioners are ideally “like a friend” for their clients, for example in their commitment to being faithfully present to them in the face of suffering (Baart 2004b). By engaging in an attentive and dedicated relationship, caregivers learn to see from an inner perspective what is at stake for their clients. The term “what is at stake” indicates that presence practitioners are sensitive to what is truly important in their clients’ lives (Baart and Vosman 2011). They draw on their professional capacity of practical wisdom to explore how the good of the client can be achieved in a specific situation (Baart 2016; Bontemps-Hommen et al. 2018; Peeters 2016; Vosman & Baart 2008). Practical wisdom acts as a moral compass, aiming to provide morally good, attuned care in the daily reality of complex care practices. This practical wisdom is gained through extensive self-reflection and deliberation with colleagues (Bontemps-Hommen et al. 2018).

To accurately identify the client’s good, presence practitioners need to shift to the client’s inner perspective. According to Baart, truly and radically taking the perspective of clients is far from self-evident in the current care and welfare system, in which the caregivers’ perspectives and gaze are guided or directed by the health system and professional standards (Baart and Grypdonck 2008). Professional caregivers tend to start from an outsider’s or external perspective and lack insight into what it is like to be on the receiving end of care: how clients experience care and what it means to them. Baart (2006a) argues that engaging with the client’s perspective requires a deliberate and radical *shift* in one’s viewing direction or orientation. He uses the concept of perspective-shifting to refer to the movement from a professional’s outer to a client’s inner perspective: the situation as it is experienced from an internal viewpoint. When practicing perspective-shifting, caregivers temporarily leave their professional point of view and move to the position of clients by standing next to them and experiencing the situation from that point of view. This shift can be both figurative and literal, as presence practitioners strive to be physically present in their clients’ lifeworld, listen attentively to their experiences, and expose themselves with all their senses to their clients’ everyday reality.

Baart, like Noddings and Meyers, does not embrace mainstream conceptualizations of empathy. In the world of health care, empathy often comes across as “soft,” therapeutic, and not radical enough. Instead, professional caregivers must truly see the other and meet people on their own terms (Baart and Goossensen 2011; Brabander 2010). Like engrossment, perspective-shifting is grounded in receptive attention. In contrast to engrossment, however, perspective-shifting primarily serves an epistemic function, aiming to gain insight into what is at stake: the client’s needs, desires, and values as seen and experienced from the client’s point of view. This insight helps caregivers to provide care that is better attuned to the individual (Baart and Grypdonck 2008).

At first glance, the concept of perspective-*shifting* may appear similar to cognitive perspective-*taking*. However, there is a notable difference. While the term perspective-taking refers to an imaginative reconstruction of the other’s experiences and situation (Oxley 2011), Baart’s perspective-shifting appears to be a more direct way of gaining insight into others. It is based on attentive perception and on being physically present in the clients’ lifeworld. Baart describes perspective-shifting as the ability to “perceive the world from a client’s perspective” [translation by first author]  (Baart 2006a, p. 743). It draws on “stories, behavior and actions, items in the house and body postures” [translation by first author] (p. 743) to grasp the essence of the other’s world. Presence practitioners are physically present in the daily reality of their clients and directly witness what people are going through. Their understandings of the other’s experiences are guided by direct perceptions and by what clients themselves show or express about their experiences and their meaning. Therefore, the concept of perspective-shifting appears to resemble the perceptive account of empathy found in Stein’s (1964) phenomenology, which can be defined as the ability to directly perceive the other’s experiences based on their gestures, facial expressions, or behavior (Meneses and Larkin 2012).

The concept of perspective-shifting is inextricably linked to the exposure, an essential and recurring practice in the presence approach. In the scientific literature, the term “exposure” refers to being subjected to aggressive substances with unknown consequences, such as chemicals, new medicines, or stressors (Baart 2006a, p. 211). In the context of the presence approach, it refers to the immersion of presence practitioners in the client’s lifeworld (Baart 2006a, p. 211). During an exposure, caregivers temporarily withhold their professional role, actions, perspective, goals, timetable, and agenda as they subject themselves attentively and with all of their senses to the client’s lifeworld. Baart describes the exposure as an exercise in being open to what is strange and alien to the caregiver (Baart 2006a; Baart and Grypdonck 2008). It is a “shift to the other, precisely in his/her otherness” [translation by first author] (Baart and Grypdonck 2008, pp. 59–60). The exposure may help caregivers to become aware of their biases and prejudices and to thoroughly get to know the client’s lifeworld and everyday reality (Baart 2006a, p. 209).

Baart emphasizes that the exposure is not only a confrontation with otherness, but also a process of self-confrontation. The exposure is expected to disrupt and affect the caregiver, as the clash of lifeworlds and perspectives “shakes up” one’s usual way of viewing (Baart 2006a). Presence practitioners who engage in an exposure try to let the environment “sink in” and reflect on the thoughts and feelings that arise, often through reflection journals, peer feedback, and supervision. This process enables them to become aware of their biases and prejudices, personal limitations, and challenges (Baart 2006a).

Baart (2006a) considers the exposure to be one of the most defining moments of the presence approach, as it is intended to contribute to a fundamental transformation process in the practice of presence. As Baart and Vosman (2011) explain, the underlying effort of the presence approach is “to reach out and make the transition from ‘being there *with*’ to ‘being there *for*,’ acknowledging that fruitful and relevant being there for someone includes an understanding from a ‘within perspective,’ that is a perspective taken within this particular relation” (p. 185). Presence practitioners focus on making themselves available rather than problem-solving. One of the main goals of the exposure is to cultivate a lasting attitude and motivation to continuously practice perspective-shifting and to radically start from the client’s perspective (Baart and Grypdonck 2008, p. 65).

## Features of care ethics empathy

In this section, we outline the differences and shared features of care ethics empathy. Each of the care ethics concepts we discussed draws on different empathic capacities and emphasizes different moral functions. Noddings’ maternal perspective draws on an affective type of empathy that primarily serves relational and motivational purposes. Meyers’ philosophical and political theory is grounded in a form of cognitive empathy that has an epistemic function. Baart’s empirically grounded presence theory draws on a perception-based type of empathy that primarily has an epistemic function. Noddings, Meyers, and Baart offer distinct insights into the aspects of empathy that must be highlighted to comprehend empathy in the field of care ethics. Their insights can elucidate how empathy contributes to good care and moral behavior, while also addressing its disadvantages and limitations. They point out aspects and qualities of empathy that have been underexposed in prevailing empathy theories, such as the relationship between empathy and receptive attention, the need for a practice of empathy that is broad in scope, or the value of being immersed in the client’s lifeworld as a means to radically shift to their perspective.

Table 1 summarizes the key features of the empathy concepts of Noddings, Meyers, and Baart.

**Table 1:** Three care ethics conceptualizations of empathy

	Noddings’ Engrossment	Meyers’ Broad Empathy	Baart’s Perspective-Shifting
Moral/caring theory in which the concept is integrated	Ethical and natural caring	Empathic thought	The presence theory
Moral question	“How would I respond to you if I were at my best caring self” (Noddings 2010b, p. 68)	“How can I best respond to you?” (Meyers 1994, p. 134)	“Who can I be for you?” (Baart and Grypdonck 2008; Baart and Vosman 2011)
Paradigmatic moral relationship	Parents and children	Acquaintances	Professional friendships
Background of the concept	Phenomenological analysis into the caregiver’s consciousness	(Feminist) philosophy and psychoanalysis	Grounded theory research into pastoral care and into a diversity of care and welfare practices
Description of the concept	Engrossment refers to a combination of receptive attention and affective empathy or “feeling with”	Broad empathy refers to the imaginative reconstruction of what it is like to be the other, ideally based on extensive interaction with that person	Perspective-shifting refers to the perceptual and mental shift from a detached outsider’s perspective to an engaged insider’s perspective
Main empathic capacity that the concept draws on	Affective: emotionally resonating or “feeling with” the other by being open and attentive to the other	Cognitive: mentally reconstructing the other’s experiences by using one’s imagination	Perceptive: perceiving what others are experiencing by opening one’s senses, being present in the clients’ lifeworld and witnessing what they are going through
Primary function(s) of the concept	Relational and motivational	Epistemic	Epistemic
Main insight into care ethics empathy that the author provides	Empathy needs to be grounded in receptive attention to help prevent self-referential strategies	To acknowledge differences and understand the meaning of the situation for a particular person, one needs to empathize with the other’s life as a whole, not only with the other’s present situation	Seeing the other’s perspective requires a deliberate and radical shift from an outsider’s to an insider’s point of view that is ideally based on an exposure into the other’s lifeworld and on being in an attentive and caring relationship
Directly related concepts	Receptive attention, motivational displacement	Receptive attention, extensive interaction	Receptive attention, exposure
How to overcome biases and prejudices?	Through ethical caring, which aims to see the other in the best possible light and which draws on critical self-reflection	Through the practice of broad empathy combined with dissident speech	Through exposing oneself to the other’s lifeworld combined with critical self-reflection
What kind of moral compass is necessary to guide empathy and move from empathy to good care or morally good actions?	The ideal of one’s “caring-best”	One’s moral identity, a dynamic, personal set of moral values that is developed in relationships with others	Practical wisdom, which can be acquired through reflecting on experiences and engaging in (moral) deliberation with colleagues

Despite the differences, the three care ethics perspectives share several fundamental features. All three authors present conceptualizations of empathy that could be conceived of as “radical empathy”, a term coined by philosopher Ratcliffe (2012). Ratcliffe’s radical empathy is characterized by a genuine commitment to engage otherness and acknowledge differences. It entails adopting a phenomenological stance of suspending one’s usual beliefs or assumptions, a willingness to be touched by the other’s experiences, and an openness to being transformed by them (Ratcliffe 2012, 2014, 2017). The three care ethics perspectives share fundamental characteristics that fit this description and that provide insights into care ethics empathy. We discuss six main features of this distinctive type of empathy.

### First, care ethics empathy is receptive and open

Receptivity is one of the most distinguishing features of care ethics empathy. Care ethicists emphasize the importance of grounding empathy in receptive attention as a means to acknowledge differences and discern others’ unique needs (Baart 2004a; Noddings 2010a; Ruddick 1989; Sevenhuijsen 2014; Tronto 1993). An important characteristic of receptive attention is that it strives to be non-judgmental and unbiased (Noddings 2010a, p. 9). Care ethics empathy entails that people try to be critically aware of their own biases, quick judgments, and assumptions and aim to bracket them (Sevenhuijsen 2014).

### Second, care ethics empathy is relational and interactive

Care ethics is a profoundly relational ethics and the type of empathy it fosters aligns with this key characteristic (Van Dijke et al. 2019, 2020). To accomplish a deep and broad engagement with the other’s experiences, care ethics empathy is ideally grounded in interpersonal interactions (Meyers 1994), preferably within a trusting relationship or encounter in which clients feel comfortable expressing themselves openly and in which they are encouraged to correct possible misunderstandings or inappropriate emotional responses (Baart 2006a). Care ethicists acknowledge that empathy and the caring relationship have a mutually reinforcing dynamic, where empathy helps to establish and strengthen the caring relationship, and the caring relationship enhances empathy.

### Third, care ethics empathy is broad and deep

instead of incident-specific and superficial. It presupposes a genuine curiosity or interest in the other and a commitment to engage with people on a deeper or broader level, for example by taking a genuine interest in the other as a whole and unique person, and by aiming to be fully present with the other. This type of empathy provides the opportunity to understand what people are going through and what values are at stake for them as seen from their perspective. It involves both the other’s experiences and what these experiences mean to that person. Thus, care ethics empathy aims to understand the other within the context of that other person’s life, values, needs, and views.

### Fourth, care ethic empathy is mature and multifaceted

Care ethicists argue that good care cannot be confined to a single perspective (Koehn 1998). Instead, they contend that multiple relevant viewpoints should be considered to determine what constitutes good care in a given situation (Baart and Vosman 2011; Meyers 1994). In the empathy literature, the ability to adopt multiple perspectives is considered a hallmark of so-called “mature empathy,” a type of higher-level or advanced empathy that involves the capacity to shift between different perspectives without losing one’s own point of view and assumes the ability to distinguish between self-experience and other-experience (Hoffman 2001; Oxley 2011). Care ethics goes a step further, emphasizing the capacity to adopt the perspective of those who are profoundly different, including people whom one may initially find offensive and unappealing, or whose values conflict with one’s own moral code (Koehn 1998; Meyers 1994; Noddings 1998).

### Fifth, care ethics empathy is critical and reflective

The care ethics alternatives discussed in this article embrace a critical perspective that acknowledges the complexities and limitations of empathy. From this standpoint, empathy should not be naively viewed as inherently good or innocent but should involve an awareness of the political and organizational context in which empathy appears, as well as a sensitivity to empathy’s power dynamics, including the risks of manipulation, paternalism, or domination. The term “critical” refers to an awareness of empathy’s challenges, particularly regarding otherness, social injustice, power, and unequal relationships (Lobb 2017). At a personal level, care professionals draw on critical self-reflection to become aware of their biases and prejudices, their preferred empathy strategies, and their personal pitfalls and limitations when practicing empathy.

### Sixth, care ethics empathy can be disruptive and transformative

 Care ethics empathy entails exposing oneself to (radical) otherness and being prepared to be emotionally affected and changed by this encounter. This form of empathy may “shake up” one’s views and bring awareness to prejudices and biases. Being affected by the other’s experiences may generate concern and strengthen one’s commitment to care (Noddings 1984/2013). Empathy has the power to broaden one’s horizon prompting people to reconsider their moral values and ideals (Meyers 1994, 2016). The empathic experience can have a lasting impact on one’s professional attitude and may fundamentally change the way one views or values the other (Baart 2006a; Meyers 1994; Noddings 1984/2013).

## Challenges of care ethics empathy

Care ethicists agree that empathy can have important moral caring functions. For example, they argue that empathy can motivate people to provide care, help to establish, maintain, and enrich caring relationships, and provide insight into what is morally at stake and needs to be addressed (Van Dijke et al. 2019). However, care ethicists also recognize that care ethics empathy, and closely related practices such as the exposure, do not necessarily guarantee good care. Noddings (1984/2013) emphasizes that engrossment alone does not always lead to a motivational displacement or caring behavior. Being engrossed can sometimes evoke feelings of revulsion or disgust, particularly towards people one does not naturally like or care for. Meyers (1994) points out that empathic understanding can be used to manipulate or control others. Baart  (2006a) cautions that people who engage in an exposure exercise may romanticize the other’s reality instead of genuinely opening up to their world.

To address these potential issues, the concept of empathy needs to be carefully embedded in a moral (caring) theory, as such theories help explain how empathy or closely related phenomena can be guided and, if necessary, corrected (Oxley 2011). In this paper, we present three ways in which moral caring theories can guide empathy. First, theories such as those presented in this paper typically consist of a constellation of related care concepts that guide empathy. For example, when empathy is anchored in receptive attention, the focus is directed towards the other instead of the self, which can help prevent self-focused empathy or projection. Concern is another key concept in relation to empathy. To contribute to good care or moral behavior, empathy needs to be grounded in concern, which can be defined as “a being willing in principle to act in such a way that this other agent will thrive” (Koehn 1998, p. 57). When people are concerned about the other’s well-being, they are less inclined to use empathy to harm or manipulate others (Meyers 1994).

Second, moral theories clarify the process that leads from the empathic experience to good care or morally good behavior. In care ethics, empathy is not primarily guided by abstract principles or rules (Koehn 1998). The three care ethicists that we discussed, criticize rule- or principle-based moral theories, such as Kantian ethics, utilitarianism, and justice theory. In general, care ethicists are suspicious of abstract or universal principles because they cannot do justice to the complexity and richness of moral situations and unique individual needs (Koehn 1998). According to them, one of the problems of rule- or principle-based moral theories is that they tend to overlook individual differences. For example, impartial reasoning draws on the golden rule question: “How would you like to be treated that way?” (Meyers 1994, p. 16). In popular parlance, this is usually understood as “treating others as you would like to be treated.” Such an understanding presupposes that the other is similar to oneself, which may lead to disregarding the other’s unique preferences, interests, and needs. The concepts of engrossment, broad empathy, and perspective-shifting are each embedded in moral theories that pose alternative moral questions (see Table 1), such as “How would I respond if I were at my best caring self?” (Noddings 2010b, p. 68), “How can I best respond to you” (Meyers 1994, p. 134), and “Who can I be for you?” (Baart and Grypdonck 2008; Baart and Vosman 2011). These questions presuppose moral subjects who engage in relationships with others and aim to meet their specific needs.

Third, additional practices and professional capacities further clarify the process of moving from empathy to moral actions or good care. For example, in Baart’s theory of presence, practical wisdom serves as a moral compass. To determine what “needs to be done”, caregivers try to understand what values are at stake for their client and reflect on what constitutes good care within the context of the larger good of the care practice of which they are a part (Vosman & Baart 2008). In Meyers’ theory, it is through a combination of one’s moral identity and broad empathic insight into the other’s values and needs that people try to figure out the best course of action. In Noddings’ care theory, it is the ideal of “one’s best caring self” that serves as a moral orientation.

Another significant challenge is the feasibility of care ethics empathy. More demanding, radical, or ambitious forms of empathy, such as those presented in this paper, may be challenging or even unattainable in everyday care settings (Meyers 1994; Ratcliffe 2012). Phrases such as “being engrossed” or “being invaded by the other” can be problematic and need to be used with caution. Without context or proper explanation, the practice of being engrossed may lead to personal distress, emotional exhaustion, or even burnout. The idea of ​​broad empathy may be hardly feasible in the context of hospital care, especially in those environments where there is little time to interact with patients, such as in the emergency room. Research indicates that empathy in health professions tends to be limited for a variety of reasons, ranging from a lack of time, few opportunities to interact with patients, the prevalence of technology over human contact, lack of empathic role models, and difficulty in relating to human suffering (Hojat 2016). Engaging otherness can be challenging for health professionals, particularly in an environment where empathy is already difficult to achieve, let alone empathizing with people who are profoundly different from the caregiver.

In addition, empathy can also be demanding for clients. Empathy is a relational practice that ideally involves client participation (Freedberg 2007; O’Hara 1997). This implies that its practice partly relies on the client’s ability and willingness to be open, vulnerable, and expressive. This is, however, far from self-evident in everyday care. Clients may resist the caregiver’s empathy, for instance, because they do not want to engage in a conversation, share personal experiences, or connect with caregivers (Agosta 2015). Additionally, clients may not be able to express themselves for a variety of reasons, including speech difficulties or trauma. Hence, care ethics empathy can be a complex and demanding practice. In the following section, we explain how care ethics empathy can still serve as an important source of inspiration for health professionals, even in light of - or perhaps because of - the challenges it entails.

## Practical implications

Despite the challenges, we argue that care ethics empathy can be a valuable addition to the field of health care. Care ethics offers an alternative and enriched conceptualization of empathy that may inspire empathy education programs and health professionals.

While the broad and deep empathy advocated by care ethics can be challenging in healthcare settings, research indicates that demonstrating a genuine interest in patients and their experiences, being open and receptive, and focusing on them as a whole person can make a significant difference in how patients feel valued and validated. For example, qualitative research into oncology patients’ perspectives on clinicians’ empathic behavior highlights the importance of active listening, empathic communication, and attending to the whole person (Sanders et al. 2021). In emergency care, it is recognized that demonstrating genuine care and being truly present with the patient instead of rushing or being distracted can help establish emergency rapport, which is particularly vital in the fast-paced context of an emergency room (Long 2017; Rosenzweig 1993). In general practice, a doctor’s ability “to develop an understanding and take into consideration what is important to the patient (that is, their beliefs, hopes, desires, and possibilities)” (p.413) is considered a fundamental element of patient-centered care. Care ethics empathy promotes this type of broad empathic engagement with the other.

In empathy education, learning about care ethics empathy may prompt students to re-examine their conventional understanding of empathy, which is often narrow and self-focused (Englander and Folkesson 2014), as opposed to a hybrid approach that embraces a broad, other-focused understanding of empathy including both affective and cognitive dimensions. Understanding empathy primarily or solely as a cognitive ability can be problematic, as the conceptualization of empathy has implications for empathy education (Jeffrey 2016). Focusing exclusively on cognitive perspective-taking, for instance, can result in education programs that teach only a limited set of mainly cognitive empathic skills and communication strategies. In contrast, a broad and hybrid understanding of empathy implies that professionals must cultivate a broader range of skills, including skills related to affective empathy, such as developing sensitivity, emotion regulation, and self-care (Ekman and Halpern 2015; Jeffrey 2016). Critics in the field of health care advocate an enriched understanding of empathy and supplementary forms of empathy education (Hanna and Fins 2006; Perrella 2016; Spiro 2016). This enhanced type of education should also embolden health professionals to genuinely engage with the patients’ experiences through interaction with actual patients and to be receptive to what people are going through. These critics argue that empathy education should be part of a comprehensive curriculum that prepares students to respond to the humanistic side of patient care (Hanna and Fins 2006; Shapiro 2008).

We assert that care ethics empathy and its education can be an important addition, as it fits the above description. With the exception of Meyers (1994), care ethicists generally advocate for a hybrid or multidimensional definition of empathy that includes the affective, cognitive, perceptive, and physical aspects of empathy (Hamington 2017; Noddings 2010b). Such a definition is in line with care ethics epistemology, which not only values cognitive knowledge sources but also embraces affective and embodied knowledge (Koehn 1998). Care ethics empathy is a receptive and relational type of empathy aiming to engage with the other’s experiences on a deeper level and to acknowledge the role and participation of the other, thereby highlighting the relational dimensions of empathy.

Various types of care ethics empathy education exist and will be explored in a future paper (Authors, forthcoming). In the Netherlands, for example, the Presence Foundation has developed an exposure exercise that is part of an extensive teaching program in presence (Baart 2006a; Baart et al. 2011). During the exercise, which is carefully prepared and supervised, caregivers temporarily step into the world of their clients and experience what life is like from that perspective. In Belgium, the care ethics lab sTimul offers a two-day exposure exercise for health professionals (Vanlaere et al. 2010, 2012). These forms of empathy education aim to cultivate a receptive type of empathy, promote awareness of personal biases and prejudices, enhance professional self-reflection, develop advanced empathic and interpersonal skills, enhance emotion regulation and self-care, and foster deep learning. It would be of great interest to consider integrating these care ethics empathy education programs into the health professions curriculum.

## Conclusion

Care ethics empathy can be defined as a multidimensional and relational form of empathy that is firmly grounded in open and receptive attention and that is radical in its commitment to engage otherness. It is an advanced or mature type of empathy that, preferably, focuses on the other’s life as a whole and strives to be physically present in the other’s lifeworld. The empathic engagement may disrupt caregivers’ reality, provoke critical reflection, and inspire them to reconsider their values and views, biases, and prejudices. Care ethics empathy may help discern clients’ unique needs and engage with their experiences on a deeper level. This radical form of empathy is both demanding and rewarding. It is our hope that care ethics empathy can help to enrich empathy education and inspire health professionals to rethink empathy, its challenges, and its significance to good care, and find encouragement and inspiration to engage otherness.
